# *HLA‐E* diversity unfolded: Identification and characterization of 170 novel *HLA‐E* alleles

**DOI:** 10.1111/tan.14195

**Published:** 2021-02-16

**Authors:** Christin Paech, Viviane Albrecht, Kathrin Putke, Gerhard Schöfl, Bianca Schöne, Alexander H. Schmidt, Vinzenz Lange, Anja Klussmeier

**Affiliations:** ^1^ DKMS Life Science Lab Dresden Germany; ^2^ DKMS Tübingen Germany

**Keywords:** genotyping, HLA‐E, next‐generation sequencing, novel allele

## Abstract

*HLA‐E* is a member of the nonclassical HLA class Ib genes. Even though it is structurally highly similar to the classical HLA class Ia genes, it is less diverse and only 45 alleles and 12 proteins were known in December 2019 (IPD‐IMGT/HLA, release 3.38.0). Since 2017, we have genotyped over 3 million voluntary stem cell donors for *HLA‐E* by sequencing the most relevant allele‐determining bases of exons 2 and 3. As expected, most donors harbor the two predominant alleles HLA‐E*01:01 and/or HLA‐E*01:03. However, in 1666 (0.05%) of our samples we detected 345 distinct novel *HLA‐E* sequences. The most frequent one was identified in 162 samples and has by now been named HLA‐E*01:114. To characterize these novel alleles in full‐length, we used both short‐read Illumina and long‐read PacBio sequencing to obtain fully phased and highly accurate sequences. This resulted in 234 submissions to IPD‐IMGT/HLA comprising 170 novel *HLA‐E* alleles, which encode for 93 novel HLA‐E proteins, as well as 64 confirmations or sequence extensions. Consequently, the number of *HLA‐E* alleles in the database (release 3.42.0) has now increased to 256 *HLA‐E* alleles and 110 HLA‐E proteins.

## 1 INTRODUCTION

*HLA‐E* is a member of the nonclassical HLA class Ib genes and is located in the major histocompatibility (MHC) complex on chromosome 6 between *HLA‐A* and *HLA‐C*. Its structure is nearly identical to the classical HLA class Ia molecules with the heavy chain pairing with an invariant beta‐2‐microglobulin light chain.^1^ However, HLA‐E only binds to a limited set of peptides containing specific anchor‐residues, which are present in the leader peptides of the classical HLA class Ia molecules.^2^ Those peptides are required for a stable HLA‐E protein and consequently for HLA‐E expression on the cellular surface.^3^ Here, HLA‐E acts as a ligand for the receptors CD94/NKG2A (inhibiting) and NKG2C (activating), which regulate NK− and CD8+ T cell activity. Due to its higher affinity to NKG2A, HLA‐E's primary role seems to be NK and CD8+ T cell‐inhibition.^4^, ^5^ This is emphasized by the fact that some viruses, for example CMV, produce HLA‐E binding peptides that might serve as an escape route from NK cell killing.^6^

The *HLA‐E* gene consists of eight exons, which encode a leader peptide (exon 1), three extracellular α domains (exons 2–4), the transmembrane region (exon 5), and the intracellular domains (exons 6–7). Exon 8 is not translated. Despite their structural and functional similarities, HLA‐E molecules are far less diverse than their classical HLA class Ia relatives. Only two different isoforms dominate in the global population, comprising a cumulative allele frequency of over 99%: HLA‐E*01:01 and HLA‐E*01:03.^7^, ^8^, ^9^ They differ in position 107 of the mature HLA‐E protein, HLA‐E*01:01 encoding for an arginine (R) and HLA‐E*01:03 for a glycine (G). Functionally, higher expression levels have been identified for G alleles.^3^ The potential impact of this dimorphism has been studied for various diseases with inconsistent results.^8^, ^10^ In kidney transplantation, HLA‐E*01:01 homozygosity of the recipient and/or donor seems to be favorable.^11^, ^12^ Recently, the dimorphism's influence on hematopoietic stem cell transplantation has been analyzed in 1840 patients. Here, a HLA‐E*01:03 homozygous donor led to a decrease in disease‐free survival and an increase in transplantation‐related mortality in patients who received T cell replete transplants.^7^ However, contradictions to other studies exist which might be caused by smaller sample sizes, different treatment regimens and other factors not yet known.^7^, ^8^, ^10^, ^13^ To our knowledge, the potential impact of alleles beside this dimorphism has never been studied.

In 2019, the IPD‐IMGT/HLA database (release 3.38.0) contained 45 *HLA‐E* alleles encoding for 12 different proteins.^14^ Since 2017, we have genotyped over 3 million newly registered potential stem cell donors for *HLA‐E* and have identified many novel sequences.^9^ In the last years, we characterized more than 3000 novel alleles for the classical HLA genes, the KIR genes, *MICA* and *MICB*.^15^, ^16^ Here, we report on the characterization of the most frequent of the so far undescribed *HLA‐E* sequences to complement the database.

## 2 METHODS

### 2.1 Sample selection

**FIGURE 1:**
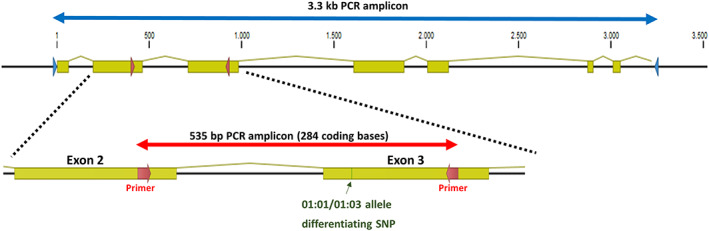
*HLA‐E* PCR amplification strategy. Arrows indicate the primer locations for the full‐gene characterization workflow (3.3 kb amplicon, blue) and high‐throughput workflow (535 bp amplicon, red). Exons are depicted as yellow boxes. The location of the HLA‐E*01:01/01:03 distinguishing SNP in exon 3 is colored in green

More than 3 million samples were genotyped for *HLA‐E* at the DKMS Life Science Lab between August 2017 and June 2020 in the course of registration as potential stem cell donors. While the majority of the donors are Europeans, other ethnicities were not excluded in this study. Consequently, it is a mixed population. In our high‐throughput workflow for HLA genotyping, which has previously been described in detail, one 535 bp amplicon, spanning the most relevant parts of exons 2 and 3, is sequenced (Figure 1).^9^, ^17^, ^18^ Genotyping data analysis is performed using the neXtype software.^17^ Sequences with mismatches to all described *HLA‐E* alleles are confirmed by an independent PCR reaction and subsequent sequencing. During the neXtype analysis, a 248 bp sequence is created digitally by deleting all intronic bases from the original PCR amplicon. These sequences were then used to count the occurrence of distinct novel sequences. To focus the full‐gene characterization process on the more abundant novel alleles, we selected three samples for each variant that we identified at least five times. In addition, we targeted less frequent variants by selecting one or two samples, resulting in a total of 273 samples. All subjects gave written informed consent in accordance with the Declaration of Helsinki. The described genotyping and extended characterization is within the scope of the consent forms signed at recruitment.

### 2.2 Full‐length PCR amplification and sequencing

For every sample, two independent PCR reactions with primers in the 3′ and 5′ UTR (fwd: CATCCGGACTCAAGAAGTTCTCAGG, rev: GAAATCCTGCATCTCAGTCGCAC) were performed to amplify a 3.3 kb fragment covering all exonic and intronic bases of HLA‐E (Figure 1). In brief, 4 μL genomic DNA was combined with 0.08 μM primer mix, 1x Advantage Genomic LA Buffer, 1.25 U Advantage Genomic LA Polymerase Mix (Takara Bio, Mountain View, California), and dNTPs (0.4 mM each) in a 25 μL reaction volume. PCR conditions: 94°C 1 minute, 35 cycles: 98°C 10 seconds/60°C 6 minutes, 72°C 10 minutes. PCR success was checked by agarose gel electrophoresis.

The product of one PCR reaction was used for Illumina shotgun sequencing. Fragmentation and adapter ligation was performed according to “NEBNext Ultra II DNA Library Prep Kit for Illumina” protocol (New England Biolabs, Ipswich, Massachusetts). After purification with 0.7x SPRIselect beads (Beckman Coulter, Brea, California), custom barcodes were attached by a 7‐cycle‐indexing PCR. Finally, 48 samples were pooled and subsequently purified using 0.7x SPRIselect beads. After qPCR library quantification, four pools were sequenced on a MiSeq instrument using a 251 bp paired‐end sequencing with MiSeq Reagent Kit v2 according to the manufacturer's instructions (Illumina, San Diego, California).

The product of the second PCR reaction was used for SMRT sequencing (Pacific Biosciences, Menlo Park, California). For this approach, the samples were barcoded by an additional 10‐cycle PCR reaction with indexing primers (0.2 μM) using indices as suggested by Pacific Biosciences. About 192 samples were then pooled and the library preparation was carried out according to manufacturer's instructions. Libraries were size selected with the BluePippin system using a 0.75% cartridge (Sage Science, Beverly, Massachusetts) and sequenced on a Sequel instrument using Sequel Sequencing Kit 3.0, SMRT Cell 1 M v3 and a 10 hours movie.

### 2.3 Data analysis

Sequencing reads obtained from Illumina and SMRT sequencing were first analyzed independently using NGSengine (GenDx, Utrecht, The Netherlands). For most samples, both methods produced a phased and consistent consensus sequence. These were approved. For the few samples with inconsistent NGSengine‐consensus sequences, we used our dual redundant reference sequencing (DR2S) pipeline, which combines short and long reads to generate highest quality error corrected consensus sequences.^15^, ^19^ Besides the targeted novel alleles, the full‐length sequences of all partner alleles were analyzed to identify additional variations. Finally, all approved sequences were submitted to IPD‐IMGT/HLA using TypeLoader2.^20^ If two identical sequences from different samples were available, the second sequence was submitted as confirmatory sequence.

### 2.4 Phylogenetic analysis

The relationship amongst 250 full‐length *HLA‐E* allele nucleotide sequences was reconstructed using a phylogenetic approach. Full length is defined as the sequence between the first base of exon 1 and the last base of exon 8. *HLA‐E* alleles with missing sequence information in this region were excluded. The best fitting model of molecular evolution (T92 + 4G^21^, ^22^) was selected based on BIC estimation as implemented in the R package DECIPHER version 2.16.1.^23^ A phylogenetic tree was constructed by maximum likelihood (ML) analysis of the nucleotide data using DECIPHER and visualized using the R package ggtree v2.2.2.^24^

## 3 RESULTS

### 3.1 Identification of novel variations in *HLA‐E* exons 2 and 3

Since the inclusion of *HLA‐E* into our high‐throughput workflow for newly registered potential stem cell donors in 2017, we have genotyped over 3 million samples for *HLA‐E*. As expected, over 99% of the observed genotypes were either HLA‐E*01:03 and/or HLA‐E*01:01.^9^ However, in 1666 samples (0.05%) we detected so far undescribed variations in the last 43 bases of exon 2 or the first 205 bases of exon 3 (Figure 1). To characterize the most frequent novel *HLA‐E* alleles, we first determined the prevalence of every distinct novel sequence in our dataset. Overall, 36 sequences were identified more than 10 times with the highest observation being 162 times. Additional 133 sequences were found at least twice and 176 novel sequences were detected only once (Table S1).

### 3.2 Full‐length characterization of novel *HLA‐E* alleles

**FIGURE 2:**
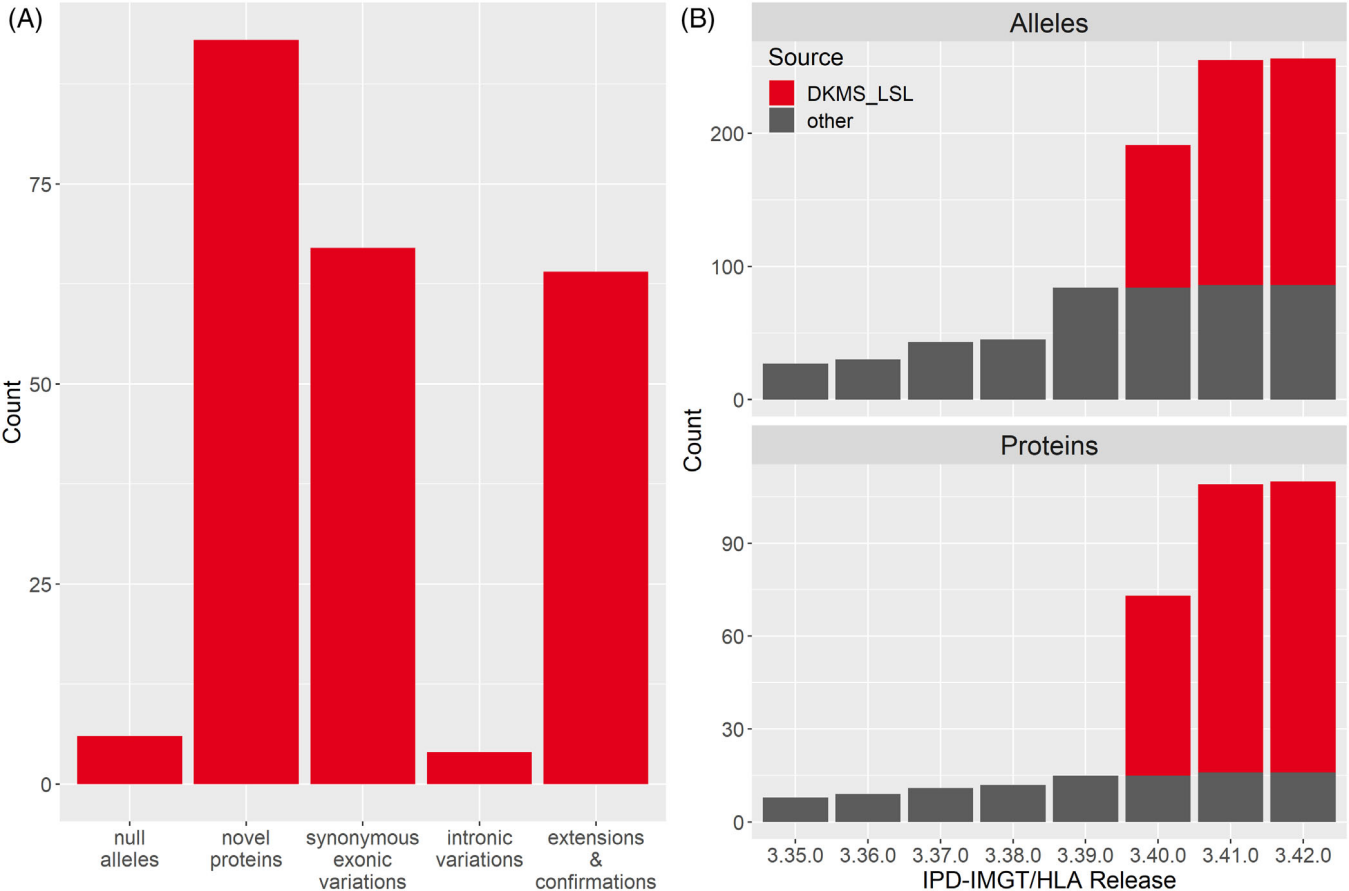
*HLA‐E* sequence submissions to IPD‐IMGT/HLA. A, Submitted *HLA‐E* sequences include 170 distinct novel alleles (null alleles, novel proteins, synonymous exonic variations, intronic variations) and 64 sequence confirmations or extensions. Sequences with more than one variation are included only once and categorized by their most relevant variation. B, Growth of the IPD‐IMGT/HLA database for *HLA‐E* alleles and HLA‐E proteins from January 2019 to October 2020. The submissions described in this study are colored in red, submissions from other laboratories are colored in gray

To characterize the identified novel *HLA‐E* sequences in full‐length, we selected 273 samples based on their observed frequency. Up to three samples were selected for each unique sequence. Every sample underwent two independent PCR reactions. One was used for short read sequencing on an Illumina instrument with high accuracy. The product from the second PCR reaction was used for long read PacBio Sequel sequencing which generates a completely phased sequence from a full‐gene amplicon.^15^ By using both sequencing methods, we were able to capitalize on the strengths of both short‐read and long‐read sequencing to determine a correct and fully phased consensus sequence for the novel allele. In 26 samples, at least one PCR reaction failed and the samples were excluded from further analysis. Reads from both sequencing methods were analyzed individually using NGSengine. For most alleles, the generated consensus sequences were identical and therefore approved immediately. All other alleles were re‐analyzed using our dual redundant reference sequencing (DR2S) pipeline, which integrates long reads and short reads to calculate consensus sequences for HLA alleles with high confidence.^15^, ^19^ The resulting high quality consensus data of 234 full‐length *HLA‐E* sequences were submitted to the IPD‐IMGT/HLA database. This corresponds to 170 novel *HLA‐E* alleles and 64 confirmatory sequences and sequence extensions (release 3.42.0). The 170 novel alleles include 93 novel HLA‐E proteins (55%), 67 alleles with synonymous variations in exons 2 or 3 (39%) and 4 alleles with intronic variations only (2%). Six novel alleles are null alleles (Figure 2A, Table 1). In cases where an identical sequence was obtained more than twice, only two sequences were submitted. Today, these newly submitted *HLA‐E* alleles represent 66% of the 256 *HLA‐E* alleles and 85% of the 110 distinct proteins (IPD‐IMGT/HLA release 3.42.0) (Figure 2B).

**TABLE 1:** Novel *HLA‐E* alleles with nonsynonymous alterations

Allele name	Reference allele	Exon	AA position (mature protein)	Reference > novel AA	AA 107	Number of observations	Frequency (%)
01:18	01:01:01:01	3	121	K > N	R	1	0.000016
01:19	01:01:01:01	2	89	E > D	R	3	0.000047
01:20	01:01:01:01	3	98	M > I	R	14	0.000219
01:21N	01:03:02:01	3	96	Q > STOP	G	9	0.000141
01:22	01:01:01:01	2	78	L > V	R	5	0.000078
01:23	01:03:02:01	2	82	R > P	G	1	0.000016
01:24	01:01:01:01	3	137	D > E	R	4	0.000063
01:25N	01:03:02:01	2	85	Y > STOP	G	4	0.000063
01:26	01:01:01:01	3	99	H > Q	R	5	0.000078
01:27	01:01:01:01	3	99	H > D	R	6	0.000094
01:28	01:01:01:01	3	111	R > H	R	3	0.000047
01:29	01:01:01:01	3	158	A > G	R	5	0.000078
01:30	01:01:01:01	3	114	E > D	R	2	0.000031
01:31	01:01:01:01	3	124	L > V	R	5	0.000078
01:32	01:01:01:01	3	112	G > R	R	28	0.000438
01:33	01:03:01:01	3	104	G > V	G	3	0.000047
01:34	01:03:02:01	3	108	R > H	G	6	0.000094
01:35	01:01:01:01	3	106	D > G	R	3	0.000047
01:36	01:01:01:01	3	104	G > R	R	4	0.000063
01:37	01:03:02:01	3	115	Q > L	G	2	0.000031
01:38	01:03:02:01	3	96	Q > P	G	27	0.000422
01:39	01:01:01:01	3	129	D > N	R	2	0.000031
01:40	01:01:01:01	3	96	Q > P	R	4	0.000063
01:41	01:01:01:01	3	105	P > S	R	16	0.000250
01:42	01:01:01:01	2	84	Y > S	R	31	0.000485
01:43	01:01:01:01	2	89	E > K	R	2	0.000031
01:44	01:03:01:01	3	131	R > S	G	2	0.000031
01:45	01:01:01:01	3	138	T > R	R	4	0.000063
01:46	01:03:02:01	3	128	E > K	G	3	0.000047
01:47	01:03:02:01	2	84	Y > H	G	18	0.000281
01:48	01:01:01:01	3	107	R > K	K	45	0.000704
01:49	01:01:01:01	2	87	Q > E	R	3	0.000047
01:50	01:01:01:01	2	83	G > D	R	2	0.000031
01:51	01:01:01:01	3	148	N > D	R	11	0.000172
01:52	01:03:02:01	3	98	M > L	G	15	0.000235
01:53	01:01:01:01	3	131	R > C	R	12	0.000188
01:54	01:03:02:01	3	102	E > Q	G	47	0.000735
01:55N	01:03:02:01	3	101	C > STOP	G	1	0.000016
01:56	01:01:01:01	2	85	Y > H	R	6	0.000094
01:57	01:03:01:01	3	106	D > E	G	2	0.000031
01:58	01:01:01:01	3	94	T > A	R	12	0.000188
01:59	01:03:02:01	3	99	H > Q	G	2	0.000031
01:60	01:03:02:01	3	140	A > V	G	6	0.000094
01:61	01:01:01:01	3	114	E > K	R	2	0.000031
01:62	01:01:01:01	3	122	D > N	R	6	0.000094
01:63	01:03:02:01	3	105	P > T	G	7	0.000109
01:64	01:01:01:01	3	137	D > Y	R	2	0.000031
01:65	01:01:01:01	3	106	D > E	R	19	0.000297
01:66	01:03:01:01	3	93	H > R	G	34	0.000532
01:67	01:01:01:01	3	110	L > V	R	3	0.000047
01:68Q	01:01:01:01	3	101	C > G	R	19	0.000297
01:69	01:03:01:01	3	95	L > P	G	2	0.000031
01:70	01:03:02:01	2	85	Y > H	G	3	0.000047
01:71	01:01:01:01	3	102	E > D	R	1	0.000016
01:72	01:01:01:01	3	99	H > Y	R	13	0.000203
01:73	01:03:02:01	3	118	Y > C	G	2	0.000031
01:74	01:01:01:01	3	138	T > A	R	3	0.000047
01:75	01:03:02:01	3	131	R > L	G	12	0.000188
01:76	01:03:02:01	3	119	D > N	G	2	0.000031
01:77	01:01:01:01	2	84	Y > H	R	23	0.000360
01:78	01:01:01:01	3	113	Y > C	R	2	0.000031
01:79	01:01:01:01	3	123	Y > F	R	1	0.000016
01:80	01:03:02:01	3	94	T > N	G	2	0.000031
01:81	01:01:01:01	3	149	D > H	R	4	0.000063
01:82	01:03:02:01	3	93	H > P	G	4	0.000063
01:83	01:01:01:01	3	141	Q > H	R	1	0.000016
01:84	01:01:01:01	3	138	T > K	R	1	0.000016
01:85	01:03:02:01	3	121	K > R	G	2	0.000031
01:86	01:03:02:01	3	108	R > L	G	1	0.000016
01:87	01:01:01:01	3	123	Y > C	R	3	0.000047
01:88	01:01:01:01	3	107	R > S	S	1	0.000016
01:89	01:03:02:01	2	83	G > S	G	2	0.000031
01:90	01:01:01:01	3	109	F > L	R	1	0.000016
01:91N	01:03:02:01	3	113	Y > STOP	G	1	0.000016
01:92	01:03:01:01	3	112	G > R	G	1	0.000016
01:93	01:03:02:01	3	92	S > Y	G	1	0.000016
01:94	01:03:02:01	2	87	Q > H	G	2	0.000031
01:95	01:03:02:01	3	156	Q > H	G	1	0.000016
01:96	01:01:01:01	3	151	S > F	R	2	0.000031
01:97	01:01:01:01	2	83	G > C	R	5	0.000078
01:98	01:01:01:01	3	151	S > P	R	1	0.000016
01:99	01:01:01:01	3	111	R > G	R	1	0.000016
01:100	01:01:01:01	2	79	R > Q	R	4	0.000063
01:101	01:03:02:01	3	91	G > V	G	1	0.000016
01:102	01:01:01:01	3	121	K > R	R	1	0.000016
01:103	01:03:01:01	2	79	R > W	G	1	0.000016
01:104	01:01:01:01	3	100	G > S	R	1	0.000016
01:105	01:01:01:01	3	102	E > A	R	1	0.000016
01:106	01:03:02:01	3	151	S > C	G	1	0.000016
01:107	01:03:01:01	3	150	A > T	G	1	0.000016
01:108	01:03:02:01	3	99	H > R	G	3	0.000047
01:109	01:01:01:01	3	125	T > A	R	1	0.000016
01:110	01:01:01:01	3	117	A > T	R	5	0.000078
01:111	01:03:02:01	3	125	T > I	G	4	0.000063
01:112	01:01:01:01	3	103	L > R	R	3	0.000047
01:114	01:01:01:01	3	94	T > S	R	162	0.002533
01:115	01:01:01:01	3	144	E > G	R	7	0.000109
01:116	01:03:02:01	2	83	G > R	G	1	0.000016
01:117N	01:01:01:01	3	96	Q > STOP	R	3	0.000047

*Notes*: Position and nature of the amino acid (AA) change are reported using the respective reference allele. AA 107 defines HLA‐E*01:01‐ or HLA‐E*01:03‐allele groups. Number of observations and frequency values are based on exon 2/3 data. Alleles are reported with two‐field resolution. The data of this table is also available as Table S2.

### 3.3 Novel *HLA‐E* alleles and phylogenetic analysis

**FIGURE 3:**
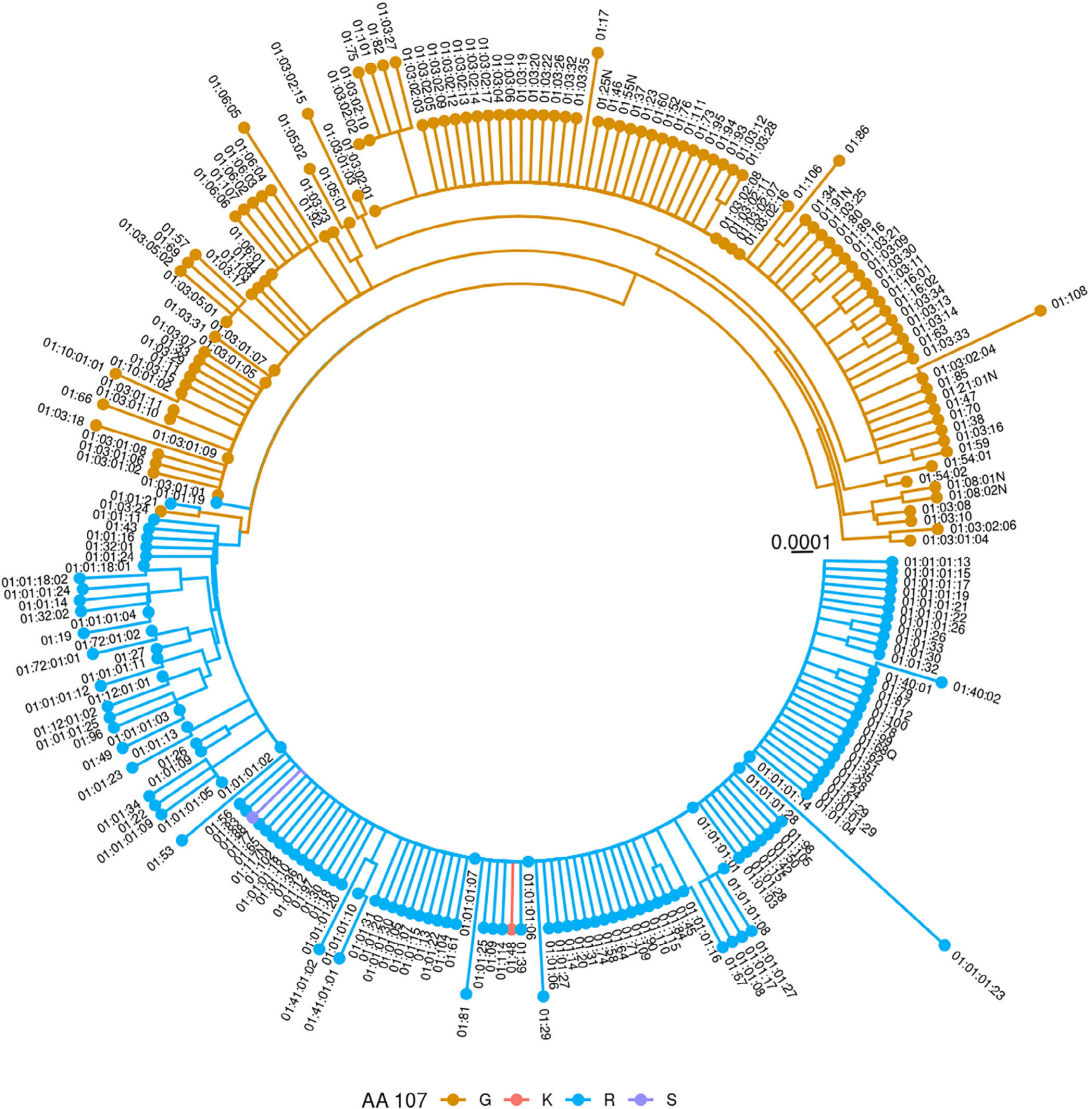
Phylogenetic tree. The 250 *HLA‐E* alleles of IPD‐IMGT/HLA release 3.42.0 with available full‐length sequence information are displayed in a maximum likelihood phylogenetic tree. Colors indicate the amino acid at position 107 of the mature HLA‐E protein

Position 107 in the mature HLA‐E protein is responsible for the common HLA‐E dimorphism: an arginine in HLA‐E*01:01 alleles and a glycine in HLA‐E*01:03 alleles. Beside this dimorphism, sequence diversity in *HLA‐E* is low, as emphasized by the generally “shallow” phylogenetic relationship amongst HLA‐E alleles. Still, the phylogenetic analysis reveals a slightly deeper branching structure for the HLA‐E*01:03 (G) alleles than for the HLA‐E*01:01 (R) alleles (Figure 3). This appears to corroborate a previous suggestion that G alleles are evolutionarily older than R alleles.^25^ Interestingly, we have identified two additional amino acid variations at position 107: a lysine was detected in 45 samples (HLA‐E*01:48, frequency 0.0007%), and a serine was detected once (HLA‐E*01:88). All other novel alleles are almost equally distributed amongst the two common variants: 90 alleles carry an arginine in position 107 while 78 alleles carry a glycine (Figure 3, Table 1). Interestingly, in five cases, we characterized the identical variation in both backgrounds: for example HLA‐E*01:47 (G) and HLA‐E*01:77 (R) or HLA‐E*01:03:24 (G) and HLA‐E*01:01:21 (R). This high similarity between HLA‐E*01:03:24 and HLA‐E*01:01:21 is the reason why HLA‐E*01:03:24 ended up in the R clade in the phylogenetic tree (Figure 3). Similar to HLA‐E*01:03:24, HLA‐E*01:01:19 is located in the middle of the two clades: it carries a synonymous variation in codon 107 (CGG instead of GGG (G alleles) or AGG (R alleles)). Furthermore, we identified six novel null alleles with HLA‐E*01:08:02N being the most frequent one (11 samples, 0.00017%). Interestingly, we never observed the previously described null allele HLA‐E*01:08:01N.^26^ The same is true for HLA‐E*01:04, whose existence has been questioned before.^10^

Overall, the newly reported *HLA‐E* alleles are rare. The most frequent allele, HLA‐E*01:114, was observed 162 times (Table 1, Table S1). This corresponds to a frequency of 0.0025% in our predominantly European sample mix. The cumulative allele frequency of all identified novel sequences is 0.026%, including sequences that have not been characterized and submitted. The limited diversity is also emphasized by the fact that most of the identified novel sequences only contained the targeted exon 2 or exon 3 mismatch. In only three cases, samples expected to be duplicates differed in an additional intron position. Only one intron variation was detected by chance in a not specifically targeted allele (HLA‐E*01:01:01:30). However, given the smaller sample size for *HLA‐E* full‐length characterization, this might not be an accurate representation of *HLA‐E* diversity across the whole gene.

## 4 DISCUSSION

Despite its high structural similarity to the classical HLA class Ia genes, the nonclassical HLA class Ib gene *HLA‐E* is much less diverse.^1^ Only 10 years ago, less than a handful of different HLA‐E proteins were known and most of the described alleles encoded the two major proteins HLA‐E*01:01 and HLA‐E*01:03. These alleles account for over 99% allele frequency in the worldwide population and are responsible for the dimorphic nature of HLA‐E.^8^ This holds also true for the 3 million potential stem cell donors we have genotyped for *HLA‐E* by NGS since 2017.^9^ Other alleles are rare. HLA‐E*01:05, which is the third most common protein encoding *HLA‐E* allele in our cohort, was identified with an allele frequency of only 0.07%. Recently, an in‐depth analysis has shown higher population frequencies (1%‐3%) in some African countries, but African populations are clearly underrepresented in our dataset.^9^ For this study, we have not systematically looked into the ethnic backgrounds or family relationships of the donors. However, for some alleles, for example, HLA‐E*01:72, we observed that most of the donors were registered with a smaller DKMS subsidiary like DKMS Chile. Consequently, it appears plausible that some of the characterized alleles may be more frequent in non‐European populations.

Our study confirms that *HLA‐E* is characterized by a much lower diversity than *HLA‐A*, *HLA‐B*, or *HLA‐C*. All identified novel *HLA‐E* alleles sum up to a cumulative allele frequency of only 0.026% in our cohort, with the most abundant allele HLA‐E*01:114 accounting for approximately one tenth of it. Therefore, it seems out of reach to observe potential functional differences in clinical studies. Nevertheless, due to our large sample throughput, we encounter novel *HLA‐E* sequences on a daily basis. As expected, the vast majority of those are extremely rare and have only been observed once or twice in the cohort. However, 36 of them have been seen at least 10 times. Consequently, we considered it worth the effort to characterize them in full‐length. This endeavor does not only reveal a broader picture of the diversity of *HLA‐E*, but also serves routine HLA genotyping, which benefits from a comprehensive allele database. Taken together, we applied *HLA‐E* full‐gene characterization on 273 samples and submitted 234 sequences to IPD‐IMGT/HLA, which include 93 novel HLA‐E proteins in 170 novel *HLA‐E* alleles as well as 64 allele confirmations or sequence extensions. Furthermore, we provide partial exon 2 and exon 3 sequences of at least 178 additional novel *HLA‐E* alleles, which were not characterized in full‐length or submitted to IPD‐IMGT/HLA due to resource prioritization (Table S1). These most certainly represent the majority of existing variations of *HLA‐E* in the European population inside the targeted region. However, the targeted region was limited to the last 43 bases of exon 2 and the first 205 bases of exon 3. Therefore, the present study is heavily biased in favor of variations inside this region. Additional variations outside this region presumably do exist.

In conclusion, we characterized 170 novel *HLA‐E* alleles in full‐length and submitted them to IPD‐IMGT/HLA. Since December 2019 (release 3.38.0), the number of known *HLA‐E* alleles has now multiplied. However, European patients harboring the described rare alleles will not be frequent. Consequently, it seems reasonable to limit *HLA‐E* genotyping to the known dimorphism in clinical studies.

## CONFLICT OF INTEREST

All authors are members of the DKMS Life Science Lab, which offers commercial genotyping services.

## AUTHOR CONTRIBUTIONS

Kathrin Putke developed and tested the primer sets. Christin Paech, Viviane Albrecht, and Bianca Schöne sequenced, analyzed and submitted the novel alleles. Anja Klussmeier selected the samples and analyzed frequency data. Gerhard Schöfl performed phylogenetic analysis. Vinzenz Lange and Anja Klussmeier wrote the manuscript. Alexander H. Schmidt, Vinzenz Lange, and Anja Klussmeier conceived and supervised the work. All authors contributed to manuscript revision, read, and approved the submitted version.

## Supporting information


Table S1

Table S2

## Data Availability

The data that support the findings of this study are available from the corresponding author upon reasonable request.
